# A prospective surveillance study of healthcare-associated infections in an intensive care unit from a tertiary care teaching hospital from 2012–2019

**DOI:** 10.1097/MD.0000000000034469

**Published:** 2023-08-04

**Authors:** Ruo-Jie Li, Yi-Le Wu, Kai Huang, Xiao-Qian Hu, Jing-Jing Zhang, Li-Qi Yang, Xi-Yao Yang

**Affiliations:** a Department of Hospital Infection Prevention and Control, The Second Affiliated Hospital of Anhui Medical University, Hefei, Anhui, China.

**Keywords:** China, device-associated infections, healthcare-associated infections, ICU

## Abstract

Healthcare-associated infections (HAIs) continue to be the most common adverse event affecting critically ill inpatients in intensive care units (ICUs). Limited data exist in the English literature on the epidemiology of HAIs in ICUs from China. The purpose of this prospective study was to understand the prevalence and trends of HAIs in the ICU to guide clinicians to take effective prevention and control measures. In total, 20 ICU beds in the hospital from January 2012 to December 2019 were selected for surveillance. HAI diagnosis and device-associated infection surveillance were based on the criteria set forth by the original Ministry of Health of the People’s Republic of China. The full-time staff for HAI management monitored all patients who stayed in the ICU > 48 hours during the study period and calculated the device utilization ratio and device-associated infection rate. The rate of HAIs and the adjusted rate were 18.78 per 1000 patient-days and 5.17 per 1000 patient-days, respectively. The rates of ventilator-associated pneumonias, catheter-associated urinary tract infections, and central line-associated bloodstream infections were 22.68 per 1000 device-days, 2.40 per 1000 device-days, and 2.27 per 1000 device-days, respectively. A total of 731 pathogenic bacteria were detected in the patients with HAIs. Gram-negative and gram-positive bacteria accounted for 67.44% and 16.83%, respectively. Continuous target monitoring, regular analysis of high-risk factors, and timely intervention measures could effectively reduce HAIs in the ICU. Additionally, these findings could be used for developing new strategies to prevent and control HAIs in ICUs.

## 1. Introduction

Healthcare-associated infections (HAIs) continue to be the most common adverse event affecting critically ill inpatients residing in intensive care units (ICUs), and the incidence of HAIs in ICUs is significantly higher than that in general wards due to the extremely vulnerable nature of patients and the frequent use of invasive procedures.^[1–4]^ HAIs could complicate the regular hospitalization process and lead to compromised patient conditions, which has been associated with increased length of hospital stay, hospitalization costs, morbidity, and mortality.^[5,6]^ The past 10 years have seen increasingly rapid advances in the field of HAI prevention and control. According to the literature,^[7,8]^ targeted surveillance was defined as the surveillance of high-risk populations and infection sites as well as risk factors among high-risk departments, such as HAI monitoring in ICUs.^[9]^ Regular infection surveillance for patients could effectively gather data on the prevalence and trends of HAIs and allow infection control professionals to define the magnitude of the problem, identify risk factors, and provide the framework for plans of intervention, all of which has become one of the crucial means of HAI management.^[10,11]^ Evidence has shown that monitoring and implementing a multidimensional approach could help decrease device-associated HAIs.^[11]^

In China, HAI incidence of patients in ICUs including surgical ICUs and general ICUs was higher than in general patients. The HAIs in ICU mainly include lower respiratory tract infection, urinary tract infection, and bloodstream infection.^[4]^ One previous study showed an increased risk of HAI in patients who with age above 80-year-old, were male, with long hospital stays, catheters, mechanical ventilation support, tracheotomy, and hemodialysis, while the use of antibiotics for preventive purposes has a protective effect.^[12]^

The International Nosocomial Infection Control Consortium, an important source of aggregate standardized international data on the epidemiology of HAIs, has acknowledged that device-associated HAI rates in limited resource countries are 3 to 5 times higher than those in Western countries.^[13]^ However, limited data exist on the epidemiology of HAIs in ICUs from China in English literature despite evidence showing global device-associated infection data in ICUs.^[14,15]^ Most studies in the field of device-associated infections have only focused on the analysis of short-term monitoring data; however, they were failed to observe trends. To further understand the trend of device-associated infections and effectively control HAIs in ICUs, this continuous 8-year HAI surveillance study in a mixed ICU with 20 beds was conducted in a tertiary care teaching hospital in China. The primary purpose of this prospective study was to determine the long-term trend in the incidence and pathogens of HAIs in 1 Chinese hospital ICU during the surveillance period to provide a reference for HAI prevention and control in ICUs.

## 2. Methods

### 2.1. Objectives

The hospital was founded in 2008 in the City of Hefei, Anhui Province of China and is a tertiary care teaching hospital with 2031 beds. It was accredited as an Academic Medical Center Hospital by Joint Commission International in January 2017. All patients admitted to the ICU for more than 48 hours were included in the study from 2012 to 2019. HAIs were defined as an infection that developed after 48 hours of mixed ICU admission and were diagnosed according to the Diagnostic Criteria for Nosocomial Infection issued by the original Ministry of Health of the People’s Republic of China,^[16]^ which were modified from the American centers for disease control and prevention (CDC).^[17]^ In the present study, infections at different sites and with different pathogens from the primary infection that occurred at least 48 hours after admission to the ICU were also classified as HAIs.^[15]^ This definition was consistent during the study period. This study was regarded as a performance improvement project of medical management and the ethical approval was not sought. Patient data were anonymized and study investigators have no access to identifying information about the patients.

### 2.2. Surveillance methods

This prospectively targeted surveillance of HAIs in the ICU was based on the standards for nosocomial infection surveillance drafted by the original Ministry of Health of the People’s Republic of China in 2009. Device-associated infection surveillance was based on the criteria required for the definition of the CDC’s national nosocomial infection surveillance system.^[17]^ Device-associated infections included ventilator-associated pneumonia, catheter-associated urinary tract infection, and central line-associated bloodstream infection.^[17]^ At least 2 to 3 times every week, the full-time staff of HAI management monitored the incidence of HAI, pathogenic microorganism, and antimicrobial agent use in the ICU. At the same time, they supervised the implementation of hand hygiene standards and other prevention and control measures of HAIs. The physician in charge of HAI surveillance diagnosed and filled out the HAI patient information form when infections occurred. The ICU patient log, which included patient admission information and diverse device utilization, was registered by an ICU nurse at a fixed time-point each day (08:00). Weekly, the resident physician gave all ICU patients a clinical condition rating according to the standard scoring system for clinical disease severity. The HAIs rates were adjusted using the patient average severity score.^[4]^

### 2.3. Statistical analysis

The total number of admitted patients, ICU-days, device-days, and patients with device-associated infections during the study period were counted.^[18]^ ICU-days were the total number of days that targeted patients were in the ICU during the specified period. Device-days were the total number of days of exposure to the devices (ventilator, urinary catheter, or central line) for targeted patients during the period. The counting data were expressed by the number of cases and percentage. Device-associated infection rates per 1000 device-days were calculated using the national healthcare safety network (NHSN) criteria for every device by dividing the device infection number by the number of device-days and then multiplying the result by 1000.^[18,19]^ SPSS 21.0 software (SPSS Inc., USA) was used for statistical analysis. The difference was detected using *χ*^2^ test. A *P* value < .05 was considered statistically significant.

## 3. Results

### 3.1. HAIs in ICU

The study included 40,262 total ICU-days from January 2012 to December 2019. A total of 756 cases of HAIs occurred. The rate of HAIs was 18.78 per 1000 patient-days. The adjusted rate of HAIs was 5.17 per 1000 patient-days based on the average illness severity of 3.63. The rate of HAIs (from 31.83 per 1000 patient-days in 2012 to 9.72 per 1000 patient-days in 2019) and the adjusted rate of HAIs (from 9.07 per 1000 patient-days in 2012 to 2.47 per 1000 patient-days in 2019) all showed a decreasing trend year-on-year from 2012 to 2019 (*P* < .001; Table 1).

**Table 1:** Incidence of healthcare-associated infections (HAIs) from January 2012 to December 2019 in the intensive care unit.

Year	Total ICU-days	No. of HAIs	Crude incidence of HAIs (%)	Rate of HAIs per 1000 patient-days (‰)	Adjusted rate of HAIs per 1000 patient-days (‰)
2012	4807	153	8.44	31.83	9.07
2013	4742	131	7.90	27.63	8.94
2014	4574	103	5.91	22.52	6.42
2015	4780	94	5.62	19.67	5.25
2016	5856	98	6.34	16.73	5.13
2017	5416	85	4.94	15.69	4.36
2018	4842	41	3.07	8.47	1.93
2019	5245	51	3.80	9.72	2.47
Total	40,262	756	5.89	18.78	5.17

HAIs = healthcare-associated infections, ICUs = intensive care units.

### 3.2. Device-associated infections

As shown in Table 2, the most frequent device-associated infection was ventilator-associated pneumonia (mean: 22.68 per 1000 ventilator-days, 478 episodes in 21,073 ventilator-days), followed by catheter-associated urinary tract infection (mean: 2.40 per 1000 urinary catheter-days, 91 episodes in 37,887 urinary catheter-days) and central line-associated bloodstream infection (mean: 2.27 per 1000 central line-days, 54 episodes in 23,830 central line-days). Statistically significant differences in the device utilization rates among ventilators, urinary catheters, and central lines were detected. The incidences of ventilator-associated pneumonia, catheter-associated urinary tract infection, and central line-associated bloodstream infection showed a downward trend from 2012 to 2019.

**Table 2:** Device utilization rate and incidence of device-associated infections from January 2012 to December 2019.

Device type	Total	2012	2013	2014	2015	2016	2017	2018	2019	χ^2^	*P* value
Total ICU-days	40,262	4807	4742	4574	4780	5856	5416	4842	5245		
Ventilator-associated pneumonia (n)	478	113	89	67	54	40	52	33	30		
* Ventilator-days*	21,073	3185	2257	2264	2252	3348	2919	2583	2265		
* Ventilator use (%*)	52.34	66.26	47.60	49.50	47.11	57.17	53.90	53.35	43.18	721.52	<.001
* VAP rate (per 1000 ventilator-days*)	22.68	35.48	39.43	29.59	23.98	11.95	17.81	12.78	13.25	98.20	<.001
Catheter-associated urinary tract infection (n)	91	24	18	6	15	11	9	2	6		
* Urinary catheter-days*	37,887	4491	4562	4336	4564	5454	4839	4646	4995		
* Urinary catheter use (%*)	94.10	93.43	96.20	94.80	95.48	93.14	89.35	95.95	95.23	334.54	<.001
* CAUTI rate (per 1000 catheter-days*)	2.40	5.34	3.95	1.38	3.29	2.02	1.86	0.43	1.20	35.60	<0.001
Central line-associated bloodstream infection (n)	54	7	3	9	6	7	11	1	10		
* Central line-days*	23,830	3421	3053	3010	3165	3666	2690	2390	2435		
* Central line use (%*)	59.19	71.17	64.38	65.81	66.21	62.60	48.67	49.36	46.43	1297.92	<.001
* CLABSI rate (per 1000 catheter-days*)	2.27	2.05	3.95	2.99	1.90	1.91	4.09	0.42	4.11	14.61	.040

CAUTI *=* catheter-associated urinary tract infection, CLABSI *=* central line-associated bloodstream infection, ICUs = intensive care units, VAP = ventilator-associated pneumonia.

### 3.3. Risk factors of HAIs

As to different subgroups, HAIs were more higher in patients who were aged > 60 years, with longer hospital stay, diabetes, surgery, central line use, and urinary catheter use (Table 3).

**Table 3:** Demographic and clinical characteristic and rates of HAIs among patients from January 2012 to December 2019 in the intensive care unit.

Variables	Total cases	No. of HAIs	HAIs rate (%)	χ2	*P* value
Age					
≤60	8981	453	5.04	34.14	<.001
>60	3850	303	7.87		
Gender					
Male	5133	329	6.41	3.67	.056
Female	7698	427	5.55		
Total length of hospital stay					
<30	8173	357	4.37	82.93	<.001
≥30	4658	399	8.57		
Use of antimicrobial agents					
Yes	12,179	730	5.99	4.06	.052
No	652	26	3.98		
Hypertension					
Yes	5774	352	6.10	0.70	.408
No	7057	404	5.72		
Diabetes					
Yes	5018	365	7.27	25.11	<.001
No	7813	391	5.00		
Surgery					
Yes	1540	252	16.36	283.73	<.001
No	11,291	504	4.46		
Ventilator (d)					
<7	9825	563	5.73	1.75	.186
≥7	3006	193	6.42		
Central line (d)					
<7	6545	244	3.73	96.10	<.001
≥7	6386	512	8.02		
Urinary catheter (d)					
<7	7162	297	4.15	78.81	<.001
≥7	5669	459	8.10		

HAIs = healthcare-associated infections.

### 3.4. Distribution of HAIs pathogens and trends in detection rate of common multidrug-resistant organisms (MDRO)

The results indicated that a total of 731 pathogen isolates were obtained from 756 cases of HAIs in the ICU (Table 4). Gram-negative bacteria predominated (67.44%), and (*Acinetobacter baumannii* [*AB*] 20.25%), (*Klebsiella pneumoniae*, 11.08%) and (*Pseudomonas aeruginosa*, 8.89%) were the most common bacteria. Gram-positive bacteria collectively accounted for 16.83%, and (*Staphyloccus aureus*, 7.66%) was the most frequent. In addition, (*Candida albicans*, 7.66%) was the most common fungus identified. The detection of common MDROs was as follows: (multidrug-resistant *Pseudomonas aerugino*, 13.32%), (carbapenem resistant *Escherichia coli*, 2.31%), carbapenem resistant (*Klebsiella pneumoniae*, 7.27%), (multidrug-resistant *Acinetobacter baumannii*, 43.48%), and methicillin resistant (*Staphylococcus aureus*, 36.15%) (Table 5).

**Table 4:** Distribution of pathogens of healthcare-associated infections from January 2012 to December 2019 in the intensive care unit.

Bacteral isolates	Total (n = 731)		2012 (n = 120)		2013 (n = 135)		2014 (n = 83)		2015 (n = 109)		2016 (n = 91)		2017 (n = 87)		2018 (n = 49)		2019 (n = 57)	
	n	%	n	%	n	%	n	%	n	%	n	%	n	%	n	%	n	%
Gram-negative bacteria	493	67.44	85	70.83	97	35.93	48	57.83	77	70.64	56	61.54	50	65.79	43	87.76	37	64.91
* P aeruginosa*	65	8.89	10	8.33	16	5.93	8	9.64	8	7.34	8	8.79	8	10.53	4	8.16	3	5.26
* E coli*	46	6.29	6	5.00	10	3.70	4	4.82	11	10.09	5	5.49	7	9.21	1	2.04	2	3.51
* K pneumoniae*	81	11.08	12	10.00	19	7.04	10	12.05	7	6.42	7	7.69	6	7.89	6	12.24	14	24.56
* Acinetobacter baumannii*	148	20.25	20	16.67	15	5.56	16	19.28	24	22.02	24	26.37	22	28.95	20	40.82	7	12.28
* stenotrophomonas maltophilia*	39	5.34	11	9.17	9	3.33	2	2.41	7	6.42	3	3.30	1	1.32	5	10.20	1	1.75
* Enterobacter cloacae*	27	3.69	10	8.33	5	1.85	3	3.61	2	1.83	3	3.30	1	1.32	0	0.00	3	5.26
* Enterobacter aerogenes*	16	2.19	4	3.33	5	1.85	1	1.20	0	0.00	3	3.30	1	1.32	1	2.04	1	1.75
* Other Gram-negative bacteria*	71	9.71	12	10.00	18	6.67	4	4.82	18	16.51	3	3.30	4	5.26	6	12.24	6	10.53
Gram-positive bacteria	123	16.83	17	14.17	23	8.52	13	15.66	17	15.60	17	18.68	24	31.58	1	2.04	11	19.30
* S aureus*	56	7.66	3	2.50	16	5.93	6	7.23	8	7.34	8	8.79	8	10.53	0	0.00	7	12.28
* Enterococcus faecalis*	7	0.96	0	0.00	0	0.00	0	0.00	1	0.92	2	2.20	3	3.95	0	0.00	1	1.75
* E faecium*	23	3.15	6	5.00	4	1.48	4	4.82	6	5.50	1	1.10	0	0.00	0	0.00	2	3.51
* Streptococcus viride*	2	0.27	1	0.83	1	0.37	0	0.00	0	0.00	0	0.00	0	0.00	0	0.00	0	0.00
* Staphylococcus haemolyticus*	10	1.37	4	3.33	0	0.00	1	1.20	2	1.83	3	3.30	0	0.00	0	0.00	0	0.00
* Other Gram-positive bacteria*	25	3.42	3	2.50	2	0.74	2	2.41	0	0.00	3	3.30	13	17.11	1	2.04	1	1.75
Fungus	115	15.73	18	15.00	15	5.56	22	26.51	15	13.76	18	19.78	13	17.11	5	10.20	9	15.79
* Candida glabrata*	26	3.56	9	7.50	3	1.11	5	6.02	1	0.92	4	4.40	4	5.26	0	0.00	0	0.00
* Candida albicans*	56	7.66	9	7.50	6	2.22	11	13.25	9	8.26	9	9.89	8	10.53	2	4.08	2	3.51
* Candida tropicalis*	21	2.87	0	0.00	3	1.11	3	3.61	4	3.67	3	3.30	1	1.32	3	6.12	4	7.02
* Aspergillus*	7	0.96	0	0.00	0	0.00	2	2.41	1	0.92	2	2.20	0	0.00	0	0.00	2	3.51
* Other fungi*	5	0.68	0	0.00	3	1.11	1	1.20	0	0.00	0	0.00	0	0.00	0	0.00	1	1.75

**Table 5:** MDRO detection rate during January 2012 to December 2019 in the intensive care unit.

MDRO	Total	2012	2013	2014	2015	2016	2017	2018	2019
MDR-PA	13.32%	20.30%	19.10%	2.10%	8.20%	6.60%	19.44%	7.10%	16.00%
CREC	2.31%	3.32%	0.00%	3.63%	3.20%	3.10%	2.27%	2.80%	1.20%
CRKP	7.27%	3.63%	3.50%	1.50%	2.88%	2.30%	12.68%	12.10%	22.70%
MDR-AB	43.48%	50.38%	69.20%	38.00%	30.60%	31.30%	38.64%	34.20%	68.20%
MRSA	36.15%	44.15%	44.90%	28.10%	37.50%	32.20%	42.31%	32.10%	25.70%

CREC = carbapenem resistant *Escherichia coli*, CRKP = carbapenem resistant *Klebsiella pneumoniae*, MDR-AB = multidrug-resistant *Acinetobacter baumannii*, MDRO = multidrug-resistant organisms, MDR-PA = multidrug-resistant *Pseudomonas aerugino*, MRSA = methicillin resistant *Staphylococcus aureus*.

## 4. Discussion

To rescue critical patients in time, an increasing number of invasive therapeutic techniques are being applied in ICUs. Ventilators, urinary catheters, and central lines are the most common invasive therapeutic instruments,^[20,21]^ all of which disrupt the body’s defense barrier and significantly increase the risk of infections.^[22]^ Device-associated HAIs pose the greatest threat to patient safety in ICUs.^[11]^

Our hospital prospectively monitored HAIs in the ICU for 8 consecutive years, and the rate of HAIs was 18.78 per 1000 patient-days. After adjustment, the rate of HAIs was 5.17 per 1000 patient-days, which was higher than the average level reported in China.^[4,23]^ The possible factors were the severe condition of ICU patients in our hospital, such as cerebral hemorrhage and multiple injuries caused by traffic accidents. Feedback on the surveillance results was provided to the ICU quarterly so that they could compare the data with earlier months/years and other hospitals to determine the potential causes and risk factors for patients with higher HAI incidence to better control the infections. In this study, the data showed a gradual decrease in HAI rate, and adjusted HAI rate over the 8 years of investigation, which showed that these infection prevention and control strategies including the improvement of facilities and enhancing compliance of hand hygiene, the strict disinfection and isolation, the establishment of infections electronic surveillance system, and constant targeted surveillance of device-associated infections, and so on in the ICU seem to have effectively reduced the risk of HAIs during that period.

The majority of HAIs in ICUs are related to the use of invasive devices.^[21]^ Therefore, this monitoring study paid particular attention to device-associated HAIs, since a significant proportion of these HAIs were considered preventable. Targeted surveillance and analysis of device-associated HAI rates per 1000 device-days standardized by the CDC’s national nosocomial infection surveillance system allows benchmarking with other similar healthcare institutions and detection of unique institutional problems.^[11]^ The results showed that the most common HAIs were ventilator-associated pneumonia, which was consistent with the previous literature.^[9]^ We observed a higher incidence of ventilator-associated pneumonia (22.68 per 1000 patient-days) than the surveillance data from other studies from China (8.89 per 1000 patient-days) and the United States (4.5 per 1000 patient-days).^[23,24]^ It may be related to the environment, economic status, medical level, and differences in severity of illness. In addition to ventilator-associated pneumonia, catheter-associated urinary tract infection (2.40 per 1000 patient-days) was the second most prevalent device-associated infection in the ICU. The rate of catheter-associated urinary tract infection was slightly higher than the surveillance data from 1 previous study in China (2.02 per 1000 patient-days)^[23]^ but less than the NHSN pooled mean (4.1 per 1000 patient-days).^[24]^ The rate of central line-associated bloodstream infection (2.27 per 1000 patient-days) was similar to that in the NHSN study (2.1 per 1000 patient-days) in the United States^[24]^ but higher than that in previous data from China (1.32 per 1000 patient-days).^[23]^ Notably, the incidence of ventilator-associated pneumonia, catheter-associated urinary tract infection, and central line-associated bloodstream infection decreased year by year from 2012 to 2019, which might be due to the benefit of implementing multiple infection control measures. In addition to some basic HAI control measures, we had implemented CDC’s Bundles to reduce device-associated infections. A series of measures such as raising the cephalothorax 30 to 45 degrees and increasing the frequency of oral care were took to prevent ventilator-associated pneumonia, some measures such as assessing the necessity of indwelling urethral catheter use daily and emptying the urine collection bag in a timely manner were took to prevent catheter-associated urinary tract infection, we took. and some measures such as using the maximum sterile barrier and enlarging the diameter of skin disinfection were took to prevent central line-associated bloodstream infection. Previous studies have shown the effectiveness of the implementation of bundles within multidimensional approaches.^[25–28]^ { INVALID CITATION,;, #2}

The risk factor analysis results of this study are similar to previous studies.^[12,18]^ Chen et al^[12]^ found that risk factors for HAI include older age (≥80 years old), male gender, length of hospital stay, and device utilization. Parajuli et al^[18]^ found that device-associated infections were highly correlated (*P* < .001) with gram-negative bacteria. A range of gram-negative organisms are responsible for device-associated infections, with the *Enterobacteriaceae* family being the most commonly identified group overall as seen in this study.^[18,29]^ A total of 731 strains of pathogenic bacteria were detected in device-related infections from 2012 to 2019 in this ICU. Gram-negative organisms accounted for 67.44%. *AB* ranked first in pathogenic bacteria for 8 aryes of surveillance (2012–2019), and the proportion trended upward year by year. Moreover, the resistance of *AB* was also notable. The drug resistance rate of multidrug-resistant *A baumannii* in our hospital was as high as 43.48%. The other common MDROs were methicillin resistant *S aureus* (36.15%), multidrug-resistant *P aerugino* (13.32%), carbapenem resistant *K pneumoniae* (7.27%), and carbapenem resistant *E coli* (2.31%). One study conducted in a Korean ICU found that the proportion of Gram-negative bacteria for device-associated infections increased from 2006 to 2014.^[30]^ The prevalence of device-associated HAIs caused by *AB* in Korean ICUs has risen rapidly, and the drug resistance rate of these bacteria to Carbapenem has also risen rapidly.^[30]^ In order to further strengthen the management of clinical application of antibiotics, the Chinese health management department has been focusing on organizing national special rectification activities for the clinical application of antibiotics in public hospitals since 2011. Our hospital has formed a multi-disciplinary antimicrobial administration group, which continuously strengthens the surveillance of antimicrobial resistance, standardizes the management of antimicrobial agents, effectively carries out the training of antimicrobial knowledge, and implements accurate infection prevention and control measures, to prevent the spread of drug-resistant bacteria. However, the trends in detection rate of MRDOs remains severe. These findings suggest that ICUs should continue to strengthen the monitoring of pathogenic bacteria, pay more attention to the changes in the distribution of pathogen isolates, and take appropriate measures to reduce the incidence of HAIs.

This study has some limitations. First, as a regional study, the results may not be representative of other ICU patients. Second, this study is retrospective and some infection cases may have been underreported. Finally, the data on infection control interventions were difficult to track back and measure during the study period, therefore the relationship between HAIs and interventions should be further explored.

## 5. Conclusion

Overall, the HAI rate was higher than the average level reported in China. The device-associated HAIs were highly correlated with gram-negative bacteria, and *AB* ranked first in pathogenic bacteria during the surveillance period. A gradual decrease in HAI rate, and adjusted HAI rate over the 8 years of investigation were detected. This prospective surveillance study suggests that continuous target monitoring, regular analysis of high-risk factors, and timely intervention measures could effectively reduce the incidence of HAIs in the ICU. Findings from this study could also be used for developing new strategies to prevent and control HAIs in ICUs.

## Acknowledgements

We thank all investigators for data collection in the intensive care unit of Second Affiliated Hospital of Anhui Medical University.

## Author contributions

**Conceptualization:** Xi-Yao Yang.

**Data curation:** Ruo-Jie Li, Yi-Le Wu, Xiao-Qian Hu, Jing-Jing Zhang.

**Formal analysis:** Ruo-Jie Li, Yi-Le Wu.

**Investigation:** Ruo-Jie Li, Yi-Le Wu.

**Methodology:** Ruo-Jie Li, Yi-Le Wu, Xiao-Qian Hu.

**Project administration:** Ruo-Jie Li, Xi-Yao Yang.

**Resources:** Ruo-Jie Li.

**Software:** Ruo-Jie Li, Yi-Le Wu.

**Supervision:** Li-Qi Yang, Xi-Yao Yang.

**Validation:** Ruo-Jie Li, Yi-Le Wu.

**Writing – original draft:** Ruo-Jie Li.

**Writing – review & editing:** Ruo-Jie Li, Yi-Le Wu, Kai Huang.
